# The validity of the selection methods for recruitment to UK core psychiatry training: cohort study

**DOI:** 10.1192/bjb.2024.9

**Published:** 2025-02

**Authors:** Paul A. Tiffin, Emma Morley, Lewis W. Paton, Nandini Chakraborty, Fiona Patterson

**Affiliations:** 1 Hull York Medical School, University of York, York, UK; 2 Work Psychology Group, Derby, UK; 3 Leicester Medical School, University of Leicester, Leicester, UK

**Keywords:** Selection, training, predictive validity, workforce, assessment

## Abstract

**Aims and method:**

Selection into core psychiatry training in the UK uses a computer-delivered Multi-Specialty Recruitment Assessment (MSRA; a situational judgement and clinical problem-solving test) and, previously, a face-to-face Selection Centre. The Selection Centre assessments were suspended during the COVID-19 pandemic. We aimed to evaluate the validity of this selection process using data on 3510 psychiatry applicants. We modelled the ability of the selection scores to predict subsequent performance in the Clinical Assessment of Skills and Competencies (CASC). Sensitivity to demographic characteristics was also estimated.

**Results:**

All selection assessment scores demonstrated positive, statistically significant, independent relationships with CASC performance and were sensitive to demographic factors.

**Implications:**

All selection components showed independent predictive validity. Re-instituting the Selection Centre assessments could be considered, although the costs, potential advantages and disadvantages should be weighed carefully.

There are chronic shortages of psychiatrists in both the UK and internationally,^1^ affecting access to mental healthcare.^2^ Selection and recruitment processes will influence the quality and quantity of practising psychiatrists. In the UK, core psychiatry training is undertaken after completion of the two Foundation Years, following graduation from medical school. Selection processes need to be fair and valid, evaluating attributes relevant to future role performance. Therefore, prior to the COVID-19 pandemic, selection for psychiatry training was nationally organised and standardised using three stages:

1. Stage 1 – Administrative: proof of eligibility for UK specialty training is checked.
2. Stage 2 – The Multi-Specialty Recruitment Assessment (MSRA), a computer-delivered test, is taken.
3. Stage 3 – The Selection Centre, involving two face-to-face assessment stations.

The MSRA has been implemented for a range of medical specialties, including psychiatry. It uses a selected response (multiple choice) question format and consists of two elements: a Situational Judgement Test (SJT, known as the Professional Dilemmas paper) and a clinical knowledge test (the Clinical Problem Solving (CPS) paper). Boxes 1 and 2 show example SJT and CPS questions.

Box 1 An example Situational Judgement Test (SJT) question

You are a Foundation (F2) doctor working in paediatrics. You are just about to leave the hospital after a long day on-call when the nurse asks you to take a telephone call from Sarah Davies. Her 3-year-old son Ben has been admitted 3 times in the past month with abdominal pain. Investigations have all been normal. Ben has the same pain again this evening. When you speak to Sarah, she wants to know why the doctors have been unable to find out what is wrong with Ben and she is concerned about his health. She is becoming upset.

*Choose the 3 most appropriate actions to take in this situation.*

- Reassure Sarah that previous investigations have been normal.
- Arrange Ben's immediate readmission to the ward.
- Arrange to repeat the investigations.
- Ask Sarah to call the GP out-of-hours service.
- Explain to Sarah that you will speak with Ben's GP the next day.
- Explain to Sarah that you will arrange a consultant review for the next clinic.
- Advise Sarah to take Ben to the Emergency Department.
- Ask Sarah more about Ben's abdominal pain.

Box 2 An example of a Clinical Problem Solving (CPS) item

A 53 year old woman with SLE presents with dull central chest pains and gradually increasing shortness of breath. Examination reveals a raised JVP, soft heart sounds and a blood pressure of 120/60, which drops to 100/60 on inspiration.

*Select the single most appropriate investigation from the list below. Select one option only.*

- Cardiac angiography
- Cardiac MRI
- ECG
- Echo
- Exercise ECG

The MSRA aims to assess the clinical and interpersonal knowledge expected of a doctor completing their Foundation Years training. Before the pandemic candidates were nationally ranked on their combined MSRA and Selection Centre scores and this determined a training place offer. From 2018 to 2020 doctors scoring above an agreed threshold (the ‘bypass score’) on the MSRA were exempt from the Selection Centre assessments. During the COVID-19 pandemic the cost/benefit ratio of maintaining face-to-face selection processes was deemed unfavourable and the Selection Centre stage was suspended. Thus, offer decisions were informed solely by the MSRA scores.

The UK-based core training of psychiatrists involves a 3-year programme with a combination of hospital and community-based placements across a variety of psychiatric specialties. During core training the trainee must pass all components of the Royal College of Psychiatrists membership (MRCPsych) examination to progress to higher specialty training. The MRCPsych has written and practical components, the latter being the Clinical Assessment of Skills and Competencies (CASC). Over the past decade or so some changes have been made to the structure of the MRCPsych (for details see the Supplementary Material, available at https://dx.doi.org/10.1192/bjb.2024.9).^3^

The CASC uses the format of an Objective Structured Clinical Examination (OSCE), having 16 stations designed to evaluate a candidate's clinical skills.^4^ The scoring system and pass standard for the CASC employs the borderline regression method.^5^ In this approach, a rater evaluates a student's performance at each station by completing a checklist and a global rating scale. The checklist marks for all examinees at each station are then regressed on the attributed global rating scores, producing a linear regression equation. The global score representing borderline performance is then substituted into the equation to predict the pass/fail score for the checklist marks. To pass the CASC a candidate must achieve a passing score in at least 12 of the 16 stations and meet or exceed the pass score set. During the COVID-19 pandemic the CASC was moved to an online format. If all elements of the MRCPsych are passed, and a candidate can evidence that the specified core competencies have been achieved, the doctor can apply for higher specialty training. Once this stage of training is completed a doctor can apply for a Certificate of Completion of Training (CCT) and be placed on the specialist register held by the General Medical Council (GMC) for the appropriate branch of psychiatry. The training process for UK psychiatry is shown in Supplementary Fig. 1.

To date there is no published research relating to the effectiveness of selection into core psychiatry training. However, the validity of the UK general practice (GP) training selection process, also using the MSRA and, until the COVID-19 pandemic, a bespoke Selection Centre, has previously been explored. The findings from these studies have raised questions about the added value of Selection Centres in GP recruitment. These reported that only a small amount of variance (around 3–4%) in the clinical GP ‘exit exam’ scores is explained by the Selection Centre ratings, once the influence of the MSRA scores is controlled for.^6–9^

Moreover, the sensitivity of selection assessments to demographic characteristics will determine the characteristics, and quantity, of the workforce selected. This is especially important in GP selection, where traditionally the workforce has had a high proportion of female, minority ethnic doctors and those who obtained their primary medical qualification outside the UK. For example, selection assessments that especially disadvantage females could have an impact on the subsequent fill rates for GP posts, in an already underserved specialty. In general, previous evidence suggests that females tend to outperform males on SJTs used for personnel selection^10^ and postgraduate clinical examinations for GP training.^11^ There is no current published evidence regarding the sensitivity of the MSRA components to candidate characteristics.

Thus, the aims of the present study were:

1. to explore the predictive validity of the differing components of the psychiatry training selection process, including among candidates with relatively low MSRA scores
2. to evaluate the impact of key demographic characteristics (world region of qualification, gender and ethnicity) on performance in the three elements of the selection process.

In relation to aim 1, the outcome of interest was the CASC score at first attempt, as a proxy for eventual clinical performance. In relation to aim 2, these analyses were intended to highlight how use of the various selection components may influence the demographics of the selected population of trainees.

## Method

### Ethics

As the study used routinely collected, de-identified data, ethical approval was not required. This was confirmed in writing by the chair of the University of York Health Sciences Ethics Committee. Moreover, it used data from the UK Medical Education Database (UKMED) and use of UKMED data is not reliant on individual consent. However, any findings published from the UKMED must be presented in blunted form.^12^ Thus, all frequencies are rounded to the nearest multiple of 5.

### Data processing and management

**Fig. 1:**
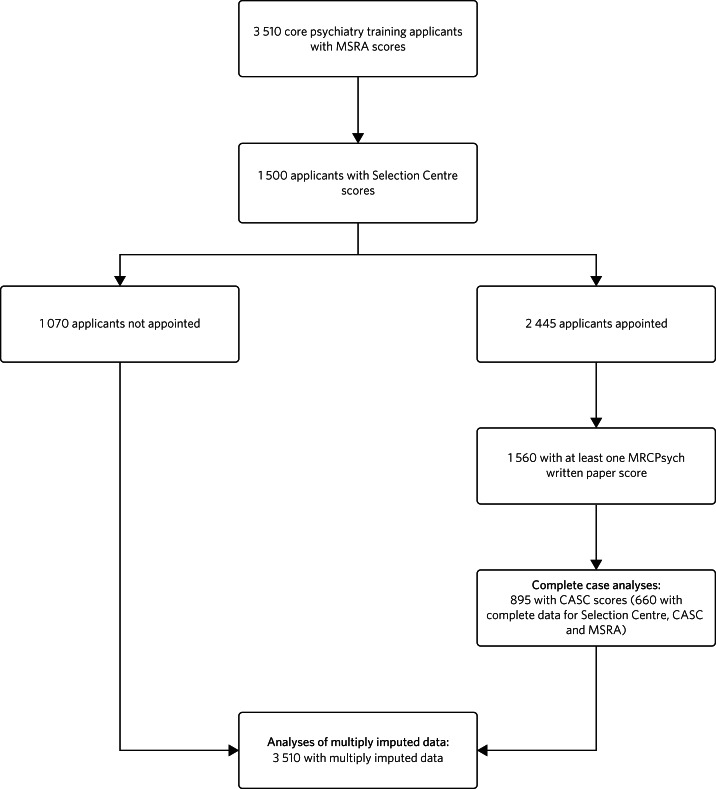
Flow of data through the study. MSRA, Multi-Specialty Recruitment Assessment; MRCPsych, Royal College of Psychiatrists membership examination; CASC, Clinical Assessment of Skills and Competencies.

Data were available from the UK Medical Education Database (UKMED) for 3510 applicants to psychiatry training with MSRA scores from the years 2015 to 2021 inclusive, with 2445 doctors appointed. The complete flow of data through the study is shown in Fig. 1.

### Measures

The SJT comprises 50 questions linked to interpersonally oriented, workplace-based scenarios. The CPS paper is made up of 97 questions using either a single best answer or enhanced matching question format, assessing medical knowledge, presenting clinical scenarios to provide context. The scores from the SJT and CPS are standardised across cohorts and summed to provide an overall MSRA score.

The Selection Centre consisted of a face-to-face ‘Presentation of Portfolio Station’ and a ‘Communications in a Clinical Setting Station’, with the scores from these summed. Details of the Selection Centre are provided in the Supplementary Material.

The outcome (CASC score) was not observable in unsuccessful applicants or those not sitting the CASC within the study time frame. This ‘range restriction’ in personnel selection studies is a special case of missing data.^13^ Consequently, missing values for both predictors and outcomes were imputed using chained equations. This is a valid approach to addressing this issue.^14–16^ As a sensitivity analysis, imputed and non-imputed results were compared (Supplementary Table 3 and Supplementary Fig. 6). To help ensure equivalence across time, the CASC scores relative to the pass mark for that sitting were used. The pass marks were not consistently available for written papers 1, 2 and 3 so the average raw scores for the first sitting of any MRCPsych written exams taken were used instead.

### Analysis approach

A series of univariable regression analyses were conducted to estimate the unadjusted relationship between the scores from the MSRA elements. The correlations between the educational assessment scores were also calculated.

The potential impact of various educational and demographic characteristics (ethnicity, gender, place of qualification) on the elements of the MSRA and the Selection Centre score were estimated. Cohen's *d* or Glass's delta were used, depending on whether or not the variances were significantly equal across groups on testing. For the Selection Centre ratings (which included imputed values owing to the bypass score system) effect sizes were derived using the miesize package for Stata 17.0 MP version for Windows.^17^ Only the selection assessment scores were entered into the multivariable model, as demographic variables are not used in selection decisions. Receiver operator characteristic (ROC) curves were generated from the imputed data, treating the MSRA scores to ‘screen’ for those passing the CASC at first attempt.

### Path analysis

**Fig. 2:**
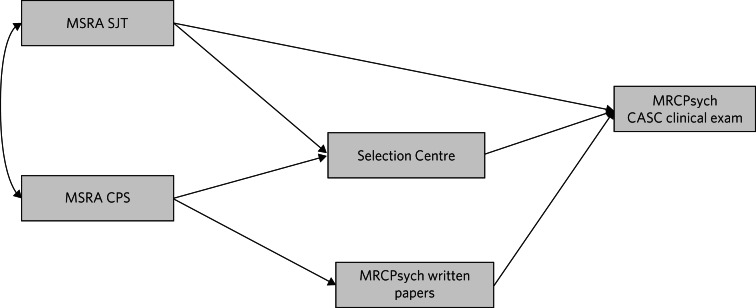
The *a priori* theoretical model hypothesising the causal relationships between the selection assessment scores and the outcome of interest (performance on the Clinical Assessment of Skills and Competencies, CASC). MSRA, Multi-Specialty Recruitment Assessment; SJT, Situational Judgement Test; CPS, Clinical Problem Solving; MRCPsych, Royal College of Psychiatrists membership examination.

Path analysis was conducted to model the unique relationships between the assessment scores and mitigate against the ‘Table 2 fallacy’.^18^ A theoretical path model for the prediction of CASC performance from the educational and selection assessment scores was developed. This was informed by prior research on predictors of postgraduate clinical exam performance^19^(Fig. 2).

The Stata and Mplus code used to manage and analyse the data is publicly available (github.com/pat512-star/P155).

## Results

A summary of the demographic and academic details for applicants and entrants are shown in Tables 1 and 2.

**Table 1:** Demographic variables for applicants to psychiatry training with and without the primary outcome of interest (CASC score at first attempt)

Demographic variable	Applicants not entering scheme	Entrants with at least one CASC attempt	All applicants	Missing values
Male gender	565/1065 (53.05%)	375/895 (41.72%)	1940/3510 (44.67%)	0/3510 (0.00%)
Non-professional SES	65/340 (18.77%)	85/530 (16.42%)	280/1590 (17.48%)	1920/3510 (54.70%)
BAME (UK graduates only)	160/465 (34.13%)	175/680 (25.59%)	620/2075 (29.90%)	85/2160 (3.89%)
Place of qualification				
UK	485/1065 (45.36%)	705/895 (82.61%)	2160/3510 (61.57%)	0/3510 (0.00%)
EEA	70/1065 (6.75%)	50/895 (5.48%)	205/3510 (5.81%)	0/3510 (0.00%)
IMG	510/1065 (47.89%)	140/895 (15.44%)	1145/3510 (32.62%)	0/3510 (0.00%)

CASC, Clinical Assessment of Skills and Competencies; SES, socioeconomic status; BAME, Black and minority ethnic; EEA, European Economic Area; IMG, international medical graduate.

**Table 2:** Educational variables for applicants to psychiatry training with and without the primary outcome of interest (CASC score at first attempt)

Educational metric	Applicants not entering scheme, mean (s.d.)	Entrants with at least one CASC attempt, mean (s.d.)
CPS score	237.32 (39.33)	251.74 (38.67)
SJT score	238.62 (39.12)	259.42 (34.93)
Selection Centre – portfolio	17.52 (8.60)	23.63 (4.28)
Selection Centre – clinical scenario	17.08 (7.48)	21.63 (3.97)
Selection Centre total score	34.60 (14.90)	45.26 (6.78)
MSRA total score	475.94 (68.90)	511.16 (64.67)
Written exam score	–	69.41 (9.05)
CASC scores (relative to pass)	–	11.36 (6.54)
CASC pass at first sitting	–	775/895 (86.80%)

CASC, Clinical Assessment of Skills and Competencies; CPS, Clinical Problem Solving; SJT, Situational Judgement Test; MSRA, Multi-Specialty Recruitment Assessment.

Applicants who entered training and had at least one attempt at the CASC recorded were more likely to be female, be a UK graduate and identify as of White ethnicity (all *P* < 0.001 on χ^2^ testing).

Please note that all the results shown here are derived from the imputed data (*m* = 10) unless otherwise indicated.

### Univariable results

#### Univariable relationship between the measures

Both components of the MSRA demonstrated significant univariable relationships with Selection Centre ratings. Similarly, the CPS and MRCPsych written examination scores were relatively highly correlated (rho = 0.52). The correlations (Spearman's rho) between the key measures are shown in Table 3 for the non-imputed data.

**Table 3:** Ranked correlations (rho values) between the metrics of interest in the non-imputed study data

Measure	CPS	SJT	Selection Centre	Written
Clinical Problem Solving (CPS)				
Situational Judgement Test (SJT)	0.43			
Selection Centre	0.28	0.34		
Written test (Written)	0.52	0.33	0.27	
Clinical Assessment of Skills and Competencies (CASC)	0.37	0.41	0.44	0.40

The results of the univariable analyses evaluating the relationship between the selection assessment and CASC scores are shown in Table 4. As can be seen, all three selection measures are significantly predictive of CASC performance.

**Table 4:** Univariable regression analyses conducted in multiply imputed data (*m* = 10) predicting Clinical Assessment of Skills and Competencies (CASC) performance (relative to the pass mark) at first attempt from scores on the three selection measures

Predictor	Coefficient (standardised β)	*P*	Lower 95% CI	Upper 95% CI
Clinical Problem Solving score	0.09 (0.47)	<0.001	0.08	0.10
Situational Judgement Test score	0.11 (0.56)	<0.001	0.10	0.13
Selection Centre total score	0.35 (0.52)	<0.001	0.29	0.41

The results of the ROC analysis are presented in the Supplementary Material.

#### Sensitivity to demographic characteristics

**Fig. 3:**
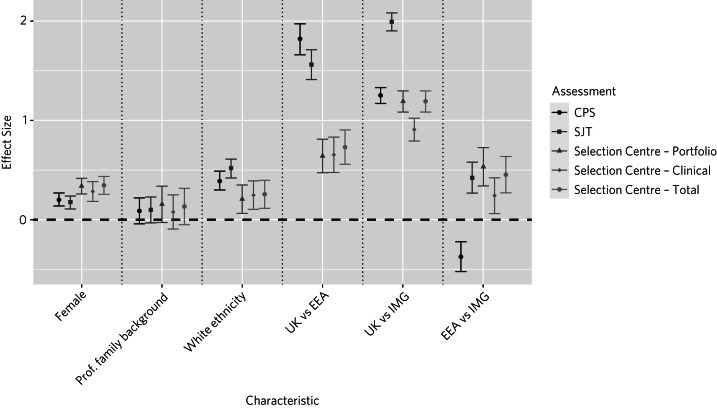
Effect sizes based on demographic characteristics. The Selection Centre effect sizes are calculated from the analysis of multiply-imputed data (*m* = 10). Socioeconomic background (professional *versus* non-professional parental occupation) were available only for UK graduates. CPS, Clinical Problem Solving; SJT, Situational Judgement Test; Prof., professional; EEA, European Economic Area; IMG, international medical graduate.

The effect sizes for the three selection measures (CPS, SJT and Selection Centre scores) are shown in Fig. 3. In general, the measures are most sensitive to world region of primary medical qualification. Compared with the MSRA elements, the Selection Centre scores were relatively insensitive to ethnicity.

### Multivariable results

The results of the multiple linear regression analysis are shown in Table 5. All the assessment scores make a significant and relatively meaningful contribution to predicting CASC performance. The predictive ability of the MSRA elements reduces, and the relative contribution of the Selection Centre scores increases, for those with low scores on the MSRA. The imputed and non-imputed data results are almost identical.

**Table 5:** Multivariable linear regression analysis predicting Clinical Assessment of Skills and Competencies (CASC) performance at first sitting from the scores from the three selection measures on the multiply imputed study data (*m* = 10)^a^

Selection assessment	Coefficient (standardised β)	*P*	Lower 95% CI	Upper 95% CI	*R*^2^ for the model
All applicants					
Clinical Problem Solving	0.04 (0.20)	<0.001	0.02	0.05	0.45
Situational Judgement Test	0.06 (0.31)	<0.001	0.05	0.08	
Selection Centre	0.23 (0.34)	<0.001	0.18	0.28	
Applicants scoring below 484 (hypothetical screening cut-off) on the MSRA					
Clinical Problem Solving	0.03 (0.13)	<0.001	0.01	0.04	0.26
Situational Judgement Test	0.06 (0.24)	<0.001	0.04	0.08	
Selection Centre	0.25 (0.39)	<0.001	0.19	0.31	

a. These results are provided for the whole sample and for those achieving relatively low scores on the Multi-Specialty Recruitment Assessment (MSRA).

### Path analysis results

A path analysis, based on the *a priori* hypothesised model (see earlier) was estimated in the imputed data-sets (*m* = 10). When evaluating model fit a confirmatory fit index (CFI) and Tucker–Lewis index (TLI) exceeding 0.90 is deemed indicative of acceptable fit, with values exceeding 0.95 consistent with ‘good’ fit.^20^

**Fig. 4:**
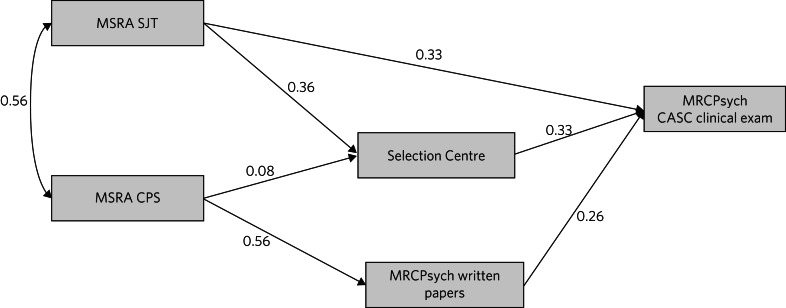
Path model A, testing the relationship between the predictors and outcome (score on the Clinical Assessment of Skills and Competencies, CASC) in the multiply imputed dataset (*n* = 3510). MSRA, Multi-Specialty Recruitment Assessment; SJT, Situational Judgement Test; CPS, Clinical Problem Solving; MRCPsych, Royal College of Psychiatrists membership examination.

**Fig. 5:**
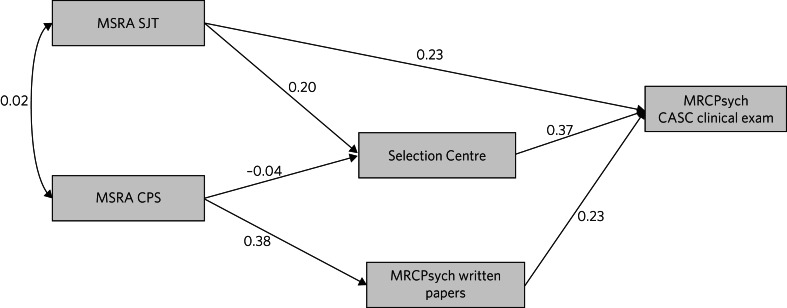
Path model B, for those scoring below 484 in the Multi-Specialty Recruitment Assessment (MSRA), using multiply imputed data (*n* = 1315). SJT, Situational Judgement Test; CPS, Clinical Problem Solving; MRCPsych, Royal College of Psychiatrists membership examination; CASC, Clinical Assessment of Skills and Competencies.

The path model for all applicants is shown in Fig. 4 and that for those with low MSRA scores (<484) is shown in Fig. 5. Both models generally showed an acceptable fit to the data (Table 6 and Figs 4 and 5). The exception was that the model for low scorers had a TLI of only 0.87. It can be seen that the relationship between the CPS and Selection Centre score is close to zero (Fig. 5). When this redundant path was omitted the TLI increased to 0.90, indicating satisfactory fit (the CFI remained at 0.96). The results of the mediational analyses are reported in the Supplementary Material.

**Table 6:** Fit indices for the two path models tested^a^

Model	d.f.	χ^2^	CFI	TLI	RMSEA
Path model for all applicants	3	130.255	0.967	0.901	0.109
Model for those scoring <484 on the MSRA	3	35.373	0.955	0.866	0.089

CFI, confirmatory fit index; TLI, Tucker–Lewis index; RMSEA, root mean square error of approximation; MSRA, Multi-Specialty Recruitment Assessment.

a. For imputed data analyses the index shown is that averaged for the results over the imputations (*m* = 10)

## Discussion

This is the first widescale study of the effectiveness of selection into UK-based core psychiatry. All three selection scores (CPS, SJT and Selection Centre) make statistically significant and independent contributions to predicting CASC performance. The relative contributions of the CPS and SJT scores were more modest for lower-scoring applicants. Conversely, the relative predictive ability of the Selection Centre scores were slightly higher in this group. Our *a priori* theoretical model was relatively well supported by the data. The exception to this was that the pathway from CPS to Selection Centre scores was weak. This is probably explained by general medical knowledge (tested by the CPS) being less related to Selection Centre performance in psychiatry.

In contrast to similar studies in GP selection^6,9,21^ we observed stronger, independent predictive effects of Selection Centre scores. This is unlikely to be due to reduced reliability of the GP Selection Centre scores: both GP and psychiatry Selection Centre assessments have moderate (0.5–0.6) reliability. Rather, it is likely that the psychiatry Selection Centres emulated elements of the CASC more closely than the GP Selection Centres relate to the Clinical Skills Assessment (the CASC equivalent).

The shortage of psychiatrists also needs to be considered. A previous study of the GP selection system highlighted that completely removing MSRA cut-off scores would increase the number of trainees more than changing the selection process in other ways. However, it would also likely increase the number of doctors failing to complete their training.^7^ Thus, the issue of an MSRA cut-off score is complex. We note that the MSRA ROC curve generated in this study was very similar to that previously reported for GP selection.^8^ Further research could investigate how selection processes relate to other important outcomes, such as retention in the practice. Further modelling, taking a Pareto-optimal perspective,^22^ could also be helpful in locating the optimum trade-off between educational performance and numbers of psychiatry trainees recruited.

With the exception of gender, the Selection Centre scores seemed less sensitive to demographic factors, including ethnicity and world region of qualification. This implies that placing some additional weight on the Selection Centre score, rather than the MSRA performance, could widen access to psychiatric training. This should be considered and further explored. However, the relative insensitivity of the Selection Centre scores to demographics may be, at least partly, an artefact of its relatively low reliability.

The present study sheds light on the process of selection into UK-based psychiatry training schemes in several important ways. First, our use of path analysis allowed a fairly nuanced understanding of the relationship between the selection assessment scores and the outcome of interest, including some of the mediational routes. Second, we were able to show the relative impact of key demographic characteristics on the selection assessment scores. The use of bypass scores resulted in Selection Centre scores that were, at times, ‘missing by design'. However, our use of missing data modelling, via multiple imputation, allowed us to derive plausible estimates for these missing Selection Centre scores, in this respect. Third, we were able to estimate our models in two subgroups of psychiatry training scheme applicants. This allowed us to evaluate whether the use of Selection Centres had a particular role in those with relatively low scores on the MSRA.

### Strengths and limitations

This was a complete national cohort of psychiatry training scheme applicants. Nevertheless, there was the obvious challenge of not being able to observe the outcome of interest in those who had been rejected, had chosen not to be appointed or had not undertaken the CASC within the study time frame. However, we were able to address this issue using multiple imputation. Our imputed and non-imputed results did not meaningfully differ, providing evidence for the validity of this approach. Ideally our validity-related outcome would have been aspects of actual workplace behaviour. However, as these were not available, high-fidelity clinical simulation examinations may be the best available proxy for this.

A key limitation is that our validity criterion was the CASC score, rather than actual workplace behaviour and practice. In this respect we cannot assume that clinical performance under the high-stakes, and somewhat artificial, CASC conditions will be consistently replicated in actual practice. However, unlike the clinical workplace and routine observer ratings, the CASC is a standardised setting, producing scores of known reliability.^23^ Moreover, actual workplace clinical practise and outcomes are challenging to capture.^24^ Thus, the CASC performance may be the best available proxy for clinical practice at present. Future research could also capture other important outcomes, such as retention in the workforce.

### Implications for policy and practice

We observed evidence of validity of the selection process. The similar coefficients for the two Selection Centre stations (clinical and portfolio) provide some support for the use of a total (summed) score. The reliability of the Selection Centre scores could have been increased by using more stations. Digitally implemented, online, interview-based approaches could also be used to reduce costs while maintaining key elements of the Selection Centre stage.^25^

The Selection Centres were contributing to the effectiveness of selection into psychiatry core training, especially for those with relatively low MSRA scores. Moreover, the Selection Centre is an opportunity to provide doctors with an early brief experience of psychiatric practice. This realistic job preview may help them decide about a psychiatry career. Thus, our findings suggest that it may be worth considering reintroducing some form of face-to-face assessment, at least for poorer (‘borderline’) performers on the MSRA. We noted that in the group with low MSRA scores, CPS and SJT scores were not correlated (Figs 4 and 5). This can be explained by the results depicted in Fig. 3. That is, the CPS and SJT show different patterns of sensitivity to world region of medical qualification, with non-UK graduates comprising the majority of applicants with relatively low MSRA scores. Thus, if a cut-off score were to be used in the future, to inform the decision of whether to offer an invitation to a Selection Centre, it may be more appropriate to apply it only to the MSRA SJT, not the CPS. A similar approach has previously been adopted in GP selection in Australia, where face-to-face processes depend on performance on an SJT.^26^

However, there are potential disadvantages to reintroducing a face-to-face Selection Centre, or equivalent. First, the use of an MSRA cut-off score creates a two-tier system and a group of candidates who may feel stigmatised by the need to pass a Selection Centre component. Second, the cost of operating Selection Centres is relatively high, and the practicalities of administration are much more demanding than relying on online testing. Unless substantial benefits were evidenced (for example, screening out unsuitable applicants) such additional burdens would be difficult to justify. Also, it may be that ‘borderline’ candidates who perform relatively poorly at a Selection Centre may respond to subsequent educational support and interventions. In this regard there is some preliminary evidence that use of ‘CASC masterclasses’ may improve the exam pass rates among non-UK medical graduates identifying as of minority ethnicity, potentially reducing differential attainment.^27^ Moreover, recently the competition ratio for core psychiatry training places in the UK has increased to a ratio of 4.98 eligible candidates per place for recruitment in 2023.^28^ In practice this means that the lowest MSRA scores where offers are being made is gradually rising.^28^ This makes a Selection Centre phase of selection less relevant, as the resultant scores contribute relatively less to predicting CASC performance in those with higher MSRA scores.

To conclude, all elements of the selection process in recruitment to core training in psychiatry appear to predict future performance in the CASC. Thus, the reintroduction of Selection Centres, for at least the poorer performers on the MSRA, could be justified. However, the costs and potential disadvantages, including unintended consequences, of Selection Centres should be considered before taking this step.

## Supporting information

Tiffin et al. supplementary material Tiffin et al. supplementary material

## Data Availability

The data used for this study are available from the UK Medical Education Database (UKMED) on application to UKMED (project number P155, with extraction generated on 29 September 2022, reissued 2 March 2023 and approved for publication on 19 May 2023). UKMED bears no responsibility for the data analysis or interpretation. The data include information derived from data collected by the Higher Education Statistics Agency Limited (HESA) and provided to the GMC (HESA Data), source: HESA Student Record 2007/2008 and 2008/2009, copyright HESA. HESA makes no warranty as to the accuracy of HESA Data and cannot accept responsibility for any inferences or conclusions derived by third parties from data or other information supplied by it. The code used to manage and analyse the data used in this study is available at github.com/pat512-star/P155.

## About the authors

**Paul A. Tiffin** is Professor of Health Services and Workforce Research and an Honorary Consultant in the Psychiatry of Adolescence in the Health Professions Education Unit, Hull York Medical School, University of York, UK. **Emma Morley** is a Chartered Occupational Psychologist and Associate Director at Work Psychology Group, Derby, UK. **Lewis W. Paton** is a Lecturer in Data Science in the Health Professions Education Unit, Hull York Medical School, University of York, UK. **Nandini Chakraborty** is a Consultant Psychiatrist and an Honorary Professor in the Department of Population Health Sciences at Leicester Medical School, University of Leicester, UK. **Fiona Patterson** is the Founding Director of Work Psychology Group, Derby, UK.
