# Supplementary material for: The validity of the selection methods for recruitment to UK core psychiatry training: cohort study
Source: BJPsych Bull. 2025 Feb;49(1):11–20. doi: 10.1192/bjb.2024.9 (PMC11810473; doi:10.1192/bjb.2024.9)
Supplement: Tiffin et al. supplementary material [file S2056469424000093sup001.docx]

**Supplementary Materials for: The validity of the selection methods for recruitment to UK core psychiatry training: a cohort study**

Paul A Tiffin, Emma Morley, Lewis W Paton, Nandini Chakraborty and Fiona Patterson

**Introduction and contents**

This *Supplementary Material* document contains additional information and results in relation to the study of the validity of the selection processes for recruitment into UK-based core psychiatry training. This additional information is useful in contextualising the findings reported in the main report.

*Detailed description of the structure of the Royal College of Psychiatrists Membership Examinations and recent changes*

During core-training the trainee must pass all components of the Royal College of Psychiatrists membership (MRCPsych) exams in order to progress to higher specialist training. The MRCPsych has written and practical components. Over the last decade or so some changes have been made to the structure of the MRCPsych (1). From March 2008, it was composed of three written exams and a practical exam, the ‘Clinical Assessment of Skills & Competencies’ (CASC). The three written exams (‘papers 1 to 3’) contained selected response questions, with both single best answer and ‘extended matching’ formats. These written tests evaluated knowledge of relevant basic science, research evidence, clinical psychiatry and medical statistics. Changes were made in 2015 so that only two written papers were taken, papers A and B. As a transitional arrangement, in order to accommodate candidates who had previously passed papers 1 or 2, paper A was split into two halves (AI and AII), covering different aspects of the MRCPsych syllabus. Paper A tests knowledge of the science and theory underpinning psychiatric practice (behavioural and social sciences, human development, neuroscience, psychopharmacology and psychiatric classification). Paper B tests knowledge of critical appraisal of research evidence and clinical topics relevant to all the psychiatric specialties.

The CASC uses the format of an Objective Structured Clinical Examination (OSCE), and is made up of two (morning and afternoon) circuits of stations designed to evaluate a candidate’s clinical skills (2). In the morning circuits, candidates have 4 minutes to read any instructions for the station and 7 minutes to complete the task. In the afternoon, this becomes 90 seconds of reading and, again, 7 minutes to complete the task. In total, there are 16 CASC stations, which test ‘history taking’, ‘examination’ (physical and mental) and ‘management’. The scoring system and pass standard for the CASC employs the borderline regression method (3). In the case of the CASC, this means that each station is marked by a trained examiner who provides two sets of scores. The first is a five-point ‘analytic’ global domain score, ranging from 1 (‘poor’) to 5 (‘excellent’) for between three and five domains. The second is a 6-point overall global judgement- ‘Excellent Pass’, ‘Pass’, ‘Borderline Pass’, ‘Borderline Fail’, ‘Fail’ or ‘Severe Fail’, which have associated grade descriptors to help anchor them. The total weighted domain scores are then regressed onto their global scores to produce a linear (regression) equation for each station for all candidates. The total domain score for borderline candidates, determined through the ‘line of best fit’ is used to set the pass mark for that station. The pass mark for the whole exam then is calculated as the average of the station pass marks for that day, with the addition of the standard error of measurement. In order to pass the CASC a candidate must achieve a passing score in at least 12 of the 16 stations and meet or exceed the overall total borderline regression score set. Thus, high scores on some stations will not necessarily compensate for low scores in others. The minimum of 12 stations as a pass standard was set on the basis that the five history-taking and five examination stations cover basic clinical skills and that a ‘borderline pass’ candidate should be expected to pass eight out of these 10 stations. Six of the stations relate to clinical management and a ‘borderline pass’ candidate was expected to pass four out of these six stations. During the Covid-19 pandemic the CASC was moved to an online format.

A description of the training pathway, and the examination system is shown in Figure S1.


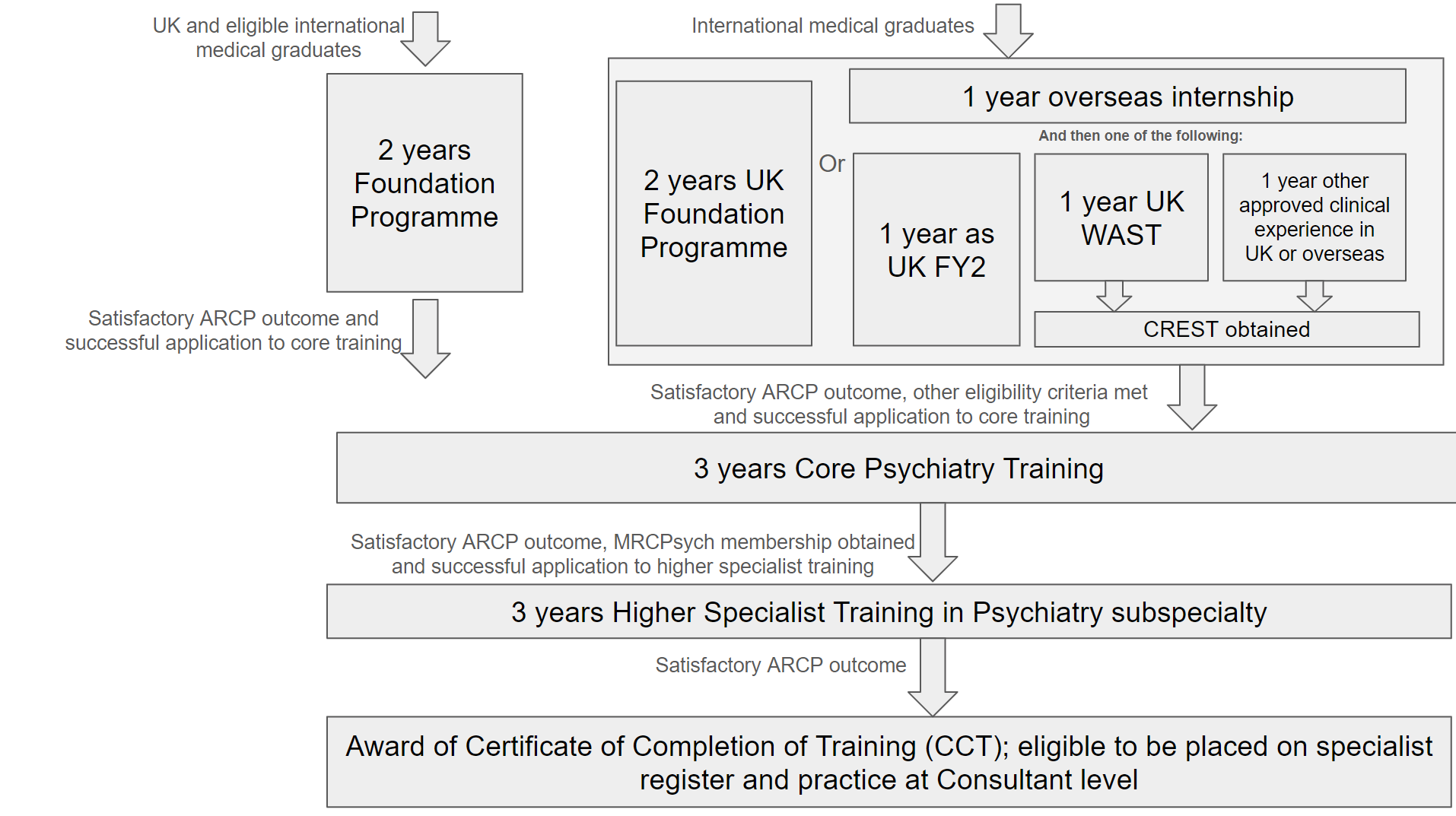


Figure S1. Flow chart of the UK psychiatry training process.

ARCP- Annual Review of Competence Progression; FY2- Second year of Foundation Programme training; CREST- Certificate of Readiness to Enter Specialty Training; MRCPsych- Membership of the Royal College of Psychiatrists obtained, involving passing all components of the MRCPsych examination and meeting the training requirements; WAST- Widening Access to Specialty Training, a scheme for overseas doctors wishing to train in the UK.

*Developing the a priori model*

This hypothesised that SC and written MRCPsych scores predict CASC performance. The CPS was conceptualised as relating mainly to future written exam performance. The SJT scores were predicted to have a direct relationship with CASC performance, as well as an indirect influence via the SC scores. This is because SJTs often yield similar levels of information to face-to-face interviews. The CPS and SJT scores were also assumed to have a relationship with the SC scores, which had both interpersonal and clinical knowledge-based components. As the CPS and SJT were sat concurrently their scores were expected to covary to some extent. This *a priori* theoretical model was then evaluated in terms of fit to the data.

*Description of the Selection Centre*

The SC consisted of a face-to-face “Presentation of Portfolio Station”, lasting 15 minutes. This assessed the domains of *commitment to specialty*, *commitment to learning and personal development*, *team working and interpersonal skills*, *academic and research skills*, *audit, teaching*, *organisational skills*, *communication skills* and *presentations skills*. This allows candidates to demonstrate achievements during foundation training (or equivalent). Portfolios usually include a CV, personal development plan, workplace-based assessments, feedback from patients or colleagues, evidence of reflective practice, quality improvement project reports and academic outputs. The second component of the SC was the “Communications in a Clinical Setting” station, also lasting 15 minutes. This aimed to evaluate *self-assurance*, *empathy*, *warmth*, *competence*, *use of active listening*, *ability to elicit history* and *reflective skills*. The first ten minutes involved an interaction with a simulated patient based on a clinical scenario. The last five minutes was a question and answer session, allowing candidates to reflect with an assessor on how the scenario went.

*Modelling the Selection Centre Scores as Separate Station Scores*

In the main report, in general, only the results for analyses using the total (summed) selection Centre (SC) scores are presented. This is because the two scores correlated at least moderately and appeared to make equal contributions to predicting the Clinical Assessment of Skills and Competencies (CASC) scores. This was because the two component scores correlated moderately (r=0.64) and exploratory modelling showed both scores had an almost identical relationship with the outcome of interest (see Figures S2 to S4). An *a priori* theoretical model was created, which included both SC station scores, and was otherwise identical to the model with a single SC total score. This is shown in Figure S2. The results of testing this model elegantly illustrated by the path diagrams in Figures S3 and S4, for the non-imputed and imputed datasets. These show the results of a path analysis which uses the clinical scenario and portfolio station scores as separate, though correlated predictor variables. As can be seen, the unique contribution to predicting the later CASC scores are identical.


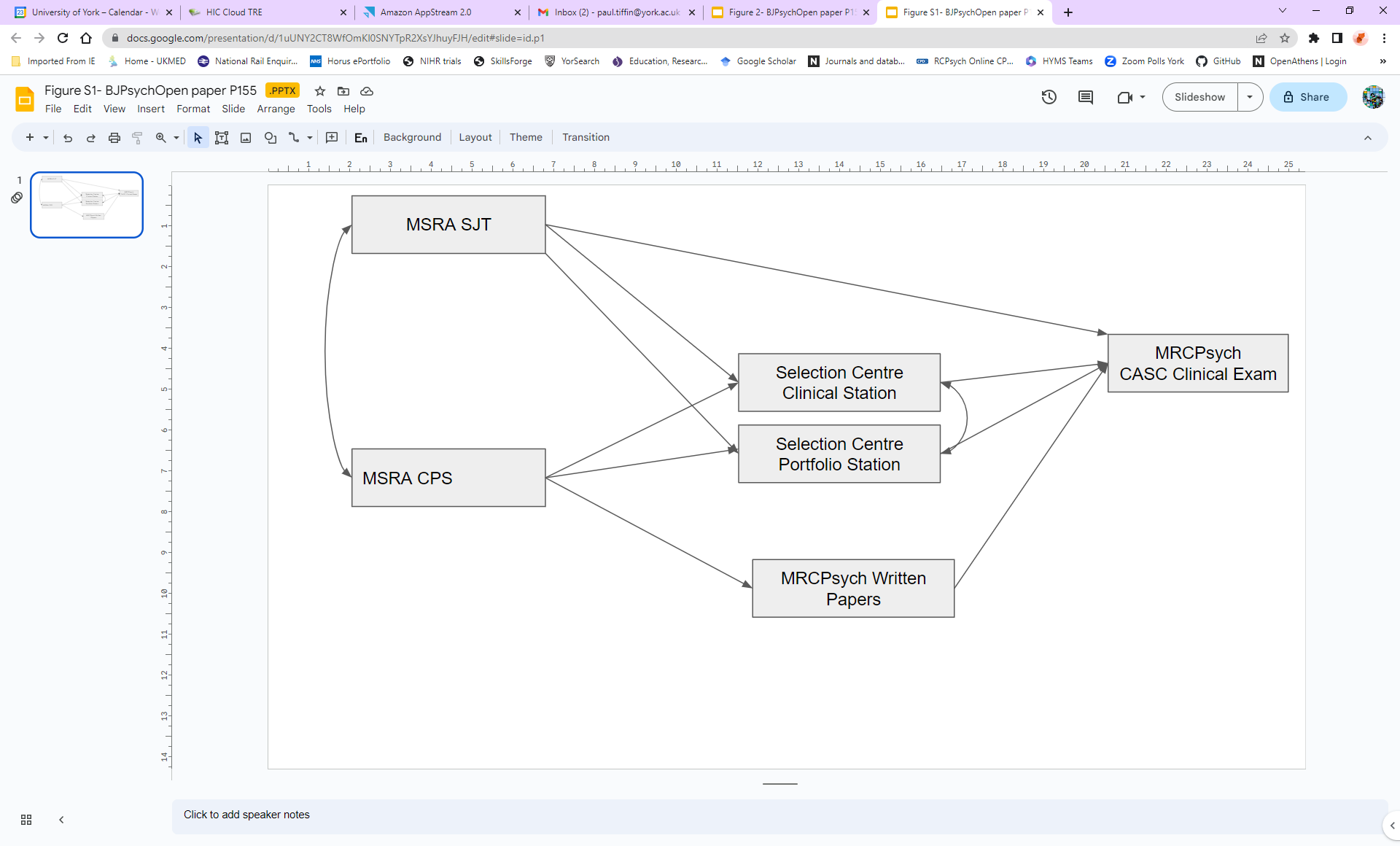


Figure S2. The *a priori* theoretical model hypothesising the causal relationships between the selection assessment scores, with separate SC station scores, and the outcome of interest (CASC performance).

MSRA- Multi-Specialty Recruitment Assessment; CPS- Clinical Problems Solving test; SJT- Situational Judgment Test


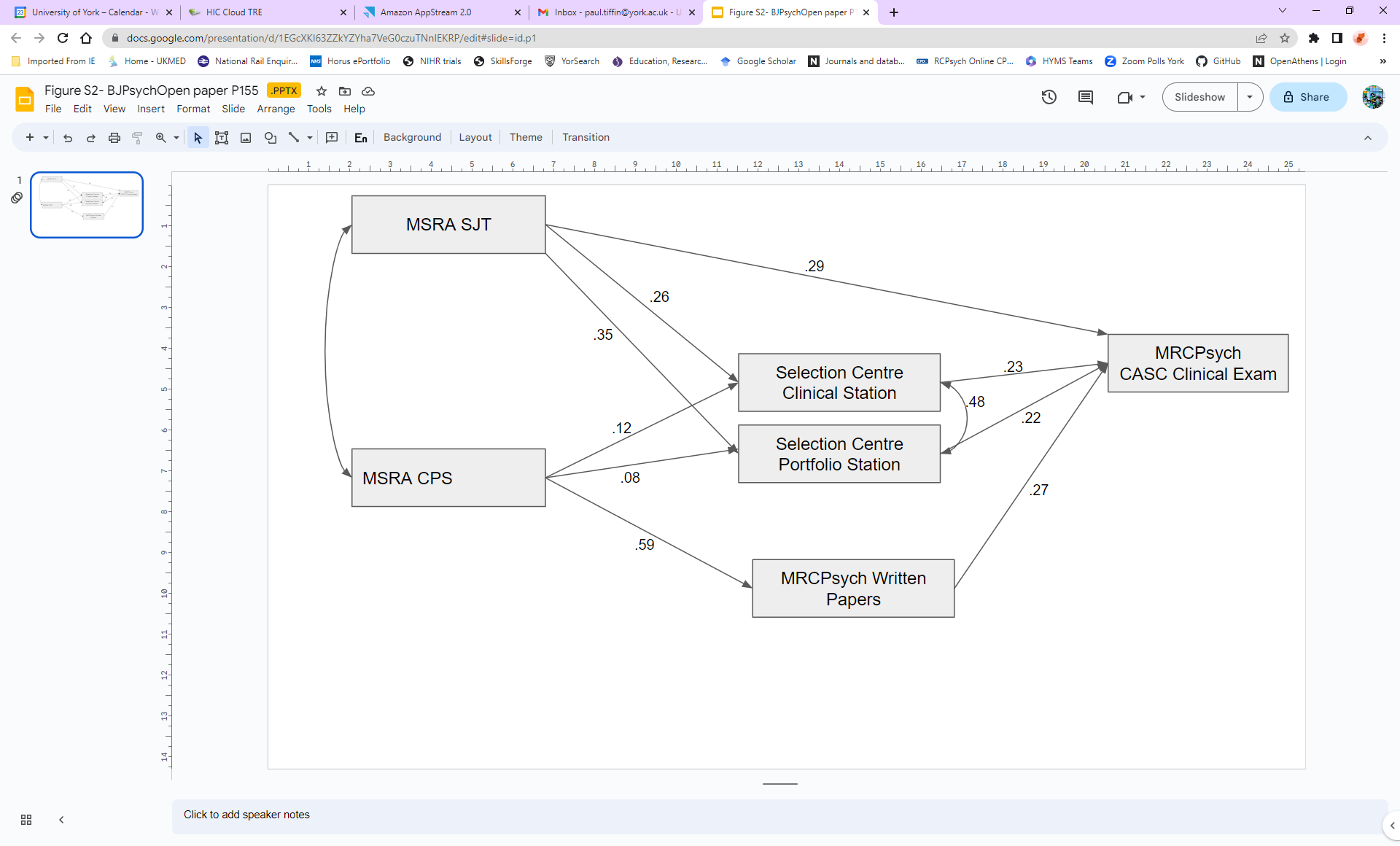


Figure S3. Results from estimating a path model with separate selection centre (SC) scores (for the clinical and portfolio (‘port’) stations respectively) testing the relationship between the predictors and outcome (CASC score) in the non-imputed dataset (N=2,015*).

MRSA- Multi-Specialty Recruitment Assessment; CPS- Clinical Problems Solving test; SJT- Situational Judgment Test

***Note that all observations, even those with some missing values, were included in the estimation using Full Information Maximum Likelihood (FIML) -except those with missing values for all the dependent variables (SC, Written and CASC, n=1,495).


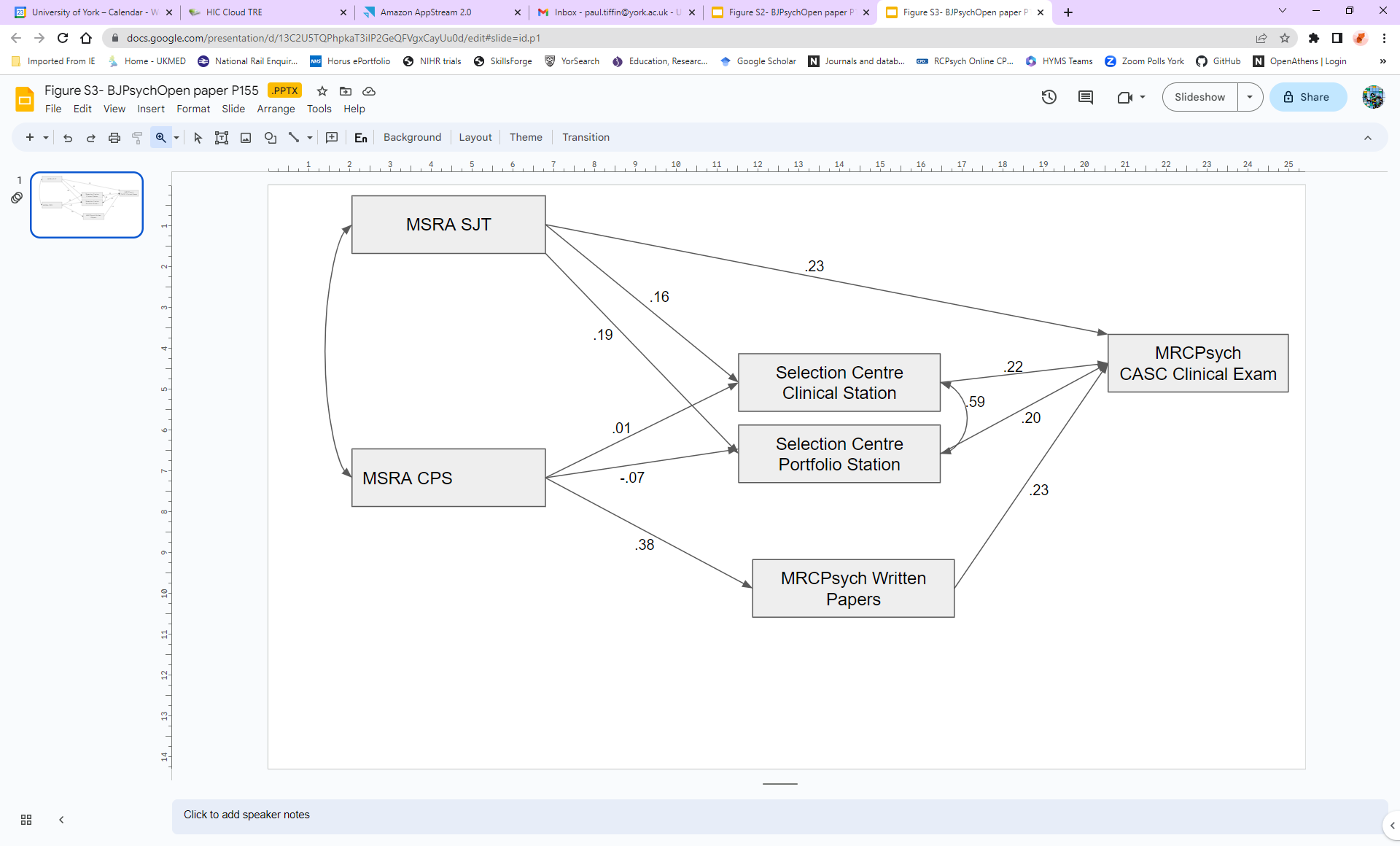


Figure S4. Path model with separate selection centre (SC) scores (for the clinical and portfolio (‘port’) stations respectively) testing the relationship between the predictors and outcome (CASC score) in the multiply imputed dataset (N=3,510).

MRSA- Multi-Specialty Recruitment Assessment; CPS- Clinical Problems Solving test; SJT- Situational Judgment Test

*Results from the Receiver Operator Characteristic (ROC) Curve Analysis*

These are the results in relation to the evaluation of the ability of the MSRA scores to act as a “screening test” when predicting passing the CASC at first attempt. The area under the receiver operator characteristic curve (AUC) was recovered and recombined from the imputed data for the MSRA predicting a CASC pass at first attempt was 0.78 (95% confidence interval 0.75 to 0.80) and was statistically significantly larger than 0.5 (p<0.001)(0.5 representing a test no better than chance). An MSRA cut-score of 484 provided an approximate optimum trade-off between sensitivity (73%) and specificity (72%). It was noted that of the 1,317 applicants that scored below this putative threshold 1,031(78%) were non-UK medical graduates (for full results see Supplementary Material; Figure S5 and Tables S1 and S2).

Figure S5. The Receiver Operator Characteristic (ROC) curve for the MSRA score ‘screening’ for an unsuccessful first attempt at the CASC. Note that this curve was recovered from imputation number ‘1’ of the study dataset.

Stata does not offer more detailed results on ROCs derived from multiply imputed data. However, the results from imputation number ‘1’ were similar to the recombined average, in terms of an AUC of 0.78. Therefore the results, in terms of specific cut-offs and the usual accuracy metrics cited in signal theory are shown below, in Table 5. Sensitivity is the ability to detect positive cases (i.e. those likely to fail the CASC at least once). Specificity is the ability to identify true negative cases (i.e. those very likely to pass the CASC at first attempt). The ‘LR+’ is the likelihood ratio for a positive test result, and an indicator of how effective the test is at detecting ‘true cases’ (i.e. candidates at genuine high risk of failing the CASC at least once). The LR+ is equivalent to the probability that a person with the ‘condition’ tested positive (i.e. a true positive) divided by the probability that a person without the ‘condition’ tested positive for it (i.e. a ‘false positive’). Conversely, the ‘LR-’ is equivalent to the probability that a person with the ‘condition’ tested negative for it (i.e. a ‘false negative’) divided by the probability that a person was a ‘true negative’. As can be seen from Table 5, a hypothetical MRSA cut-score of 484 provides a balance (trade-off) between sensitivity and specificity, with both exceeding 70%.

| Cut-point | Sensitivity | Specificity | Correctly classified | LR+ | LR- |
| --- | --- | --- | --- | --- | --- |
| >=265 | 99.93% | 0.12% | 76.89% | 1.0005 | 0.6000 |
| >=340 | 99.26% | 5.43% | 77.61% | 1.0496 | 0.1364 |
| >=400 | 95.26% | 23.95% | 78.80% | 1.2526 | 0.1979 |
| >=450 | 84.85% | 52.72% | 77.44% | 1.7945 | 0.2874 |
| >=484 | 72.81% | 71.98% | 72.62% | 2.5982 | 0.3777 |
| >=500 | 65.37% | 79.63% | 68.66% | 3.2091 | 0.4349 |
| >=550 | 32.11% | 93.33% | 46.24% | 4.8167 | 0.7274 |
| >=600 | 4.56% | 99.01% | 26.35% | 4.6125 | 0.9640 |

Table S1. The ability of the MSRA to “screen out” candidates at high risk of failing the CASC at first attempt, for different example hypothetical cut-scores. Note these metrics were produced from an analysis of imputed dataset number ‘1’.

However, it is important to appreciate what the absolute numbers of passes and fails at first CASC attempt are likely to be if the MSRA was used as a screening test for this purpose. Therefore a confusion matrix (two by two contingency table) was created from the imputed outcomes. For those without observed CASC attempts a “pass” was imputed where the predicted probability of passing the examination at first attempt was estimated to be equal to or exceed 0.5 (i.e. the average pass (1) or fails (0) in the 10 imputed datasets averaged 0.5 or more). Likewise, if this was estimated to be less than 0.5 an overall fail (0) was imputed. The resulting confusion matrix is shown in Table S2.

As can be seen from Table S2, the overall accuracy of this MSRA cut-off is 74% with 2,600 of applicants correctly classified. The positive predictive value (PPV) is 96% (2,095/2,180). That is, of those predicted to pass at first attempt 96% actually do so. The negative predictive value (NPV) is less impressive at 38%, with this being the proportion of applicants predicted to fail the CASC at first attempt actually doing so.

| **Prediction**  **(Above MSRA cut-point of 484 points)** | **Actual/imputed fail?** | **Actual/imputed pass?** | **Total** |
| --- | --- | --- | --- |
| No- predicted to fail CASC at 1^st^ attempt | 505 | 830 | 1,330 |
| Yes- predicted to pass CASC at 1^st^ attempt | 80 | 2,095 | 2,180 |
| Total | 585 | 2,925 | 3,510 |

Table S2. A confusion matrix depicting the performance of the MSRA as a screening test to predict the likelihood of passing the CASC at first attempt. The estimates were derived from the imputed data (M=10, N=3,510)

The authors of a similar study in recruitment to General Practice training suggested that the optimum cut-off for the MSRA would be around 500, given this ROC (4). This would also be true for psychiatry applicants, if one considers a reasonable trade-off between sensitivity and specificity (see Supplementary Material; Figure S4 and Table S1). However, given the costs and benefits of having vacant consultant (or other) psychiatric posts, versus core trainees failing to complete training etc selectors may wish to optimise for specificity rather than sensitivity. Thus, the issue of an optimum cut-off score, or indeed entire selection system is a complex one, as previously highlighted in relation to recruitment to GP training schemes (5). Certainly, the results shown in our confusion matrix (Table S2) illustrate what the relatively high PPV and low NPV of the MSRA as a screening test for passing the CASC at first attempt may mean in absolute numbers. That is, of those 1,300 or so applicants scoring less than the hypothetical MSRA cut-off, more than half would still be likely to pass the CASC at first attempt. Moreover, it may be that many of the applicants failing the CASC would pass at second, or third attempt. Consequently, using the MSRA as a sole threshold criteria for admission to core psychiatry training may be undesirable, given it is a medical specialty that has significant workforce shortages. Moreover, it may also be useful to take a ‘pareto-optimal’ approach to selection, in order to use the various selection measures to model the more and less desirable combinations of trade-offs between fill rates and likelihood of passing licensure exams in a timely way (6).

*Additional results from non-imputed data*

In order to evaluate the extent to which the missing at random (MAR) assumption was upheld, we compared the findings from the non-imputed and imputed data. In Table S3 we include the results from non-imputed results for the multivariable linear regression. As expected, the correlations between the predictors and outcomes of interested tended to be modestly higher in the results from the imputed data. This is to be expected as the approach is designed to counter the apparent attenuation of these relationships that occurs due to ‘restriction of range’ in personnel selection studies. This occurs as only those selected, or having a work related outcome observed within a study timeframe, will have data relating to the primary outcome of interest. This artificially reduces the observed correlation between a predictor and validity criterion, which, previously, led selectors to erroneously conclude that a selection assessment was invalid. Note that for the SC scores, the raw β coefficient is slightly larger in the non-imputed data, compared to the imputed data. However the reverse is true for the standardised coefficient for the effect of SC score on CASC performance (see Table S3). This apparent paradox is assumed to be due to the metric (distribution) of the predictor and outcome of interest differing between the imputed and non-imputed data. Specifically this effect is almost certainly mainly due to the relatively increased variance in the imputed CASC scores, compared to non-imputed.

| **Selection assessment** | **Coefficient (β)** | **p** | **lower 95% CI** | **upper 95% CI** | **R^2^ for the model** |
| --- | --- | --- | --- | --- | --- |
| **All applicants** | | | | |  |
| Clinical Problem Solving | .04 (.20) | <0.001 | 0.02 | 0.05 | 0.45 |
| Situational Judgement Test | .06 (.31) | <0.001 | 0.05 | 0.08 |  |
| Selection Centre | .23 (.34) | <0.001 | 0.18 | 0.28 |  |
| **Applicants scoring below 484 (hypothetical screening cut-off) on the MSRA** | | | | | |
| Clinical Problem Solving | 0.03 (0.13) | <0.001 | 0.01 | 0.04 | 0.26 |
| Situational Judgement Test | 0.06 (0.24) | <0.001 | 0.04 | 0.08 |  |
| Selection Centre | 0.25 (0.39) | <0.001 | 0.19 | 0.31 |  |
| **Results from non-imputed data** | | | | | |
| Clinical Problem Solving | 0.03 (0.20) | <0.001 | 0.02 | 0.05 | 0.34 |
| Situational Judgement Test | 0.05 (0.26) | <0.001 | 0.03 | 0.06 |  |
| Selection Centre | 0.29 (0.31) | <0.001 | 0.23 | 0.35 |  |

Table S3. Results from multivariable linear regression analysis predicting CASC performance at first sitting from the scores from the three selection measures (CPS, SJT and SC) on the multiply imputed study data (m=10). These results are provided for the whole sample and for those achieving relatively low MSRA scores. The last three rows also report the results from analysis of the non-imputed data.

For the path analyses, when using the non-imputed data, we applied “full information maximum likelihood” (FIML) as the estimation approach in Mplus. FIML also handles missingness based on the MAR assumption. However, this approach excludes a proportion of observations with missing values for *all* dependent variables in the path model. In this case these are the SC, written examination and CASC scores which were completely missing for 1,495 doctors, representing 43% of the total data. The results from the non-imputed datasets are presented here so they can be compared as a sensitivity analysis.


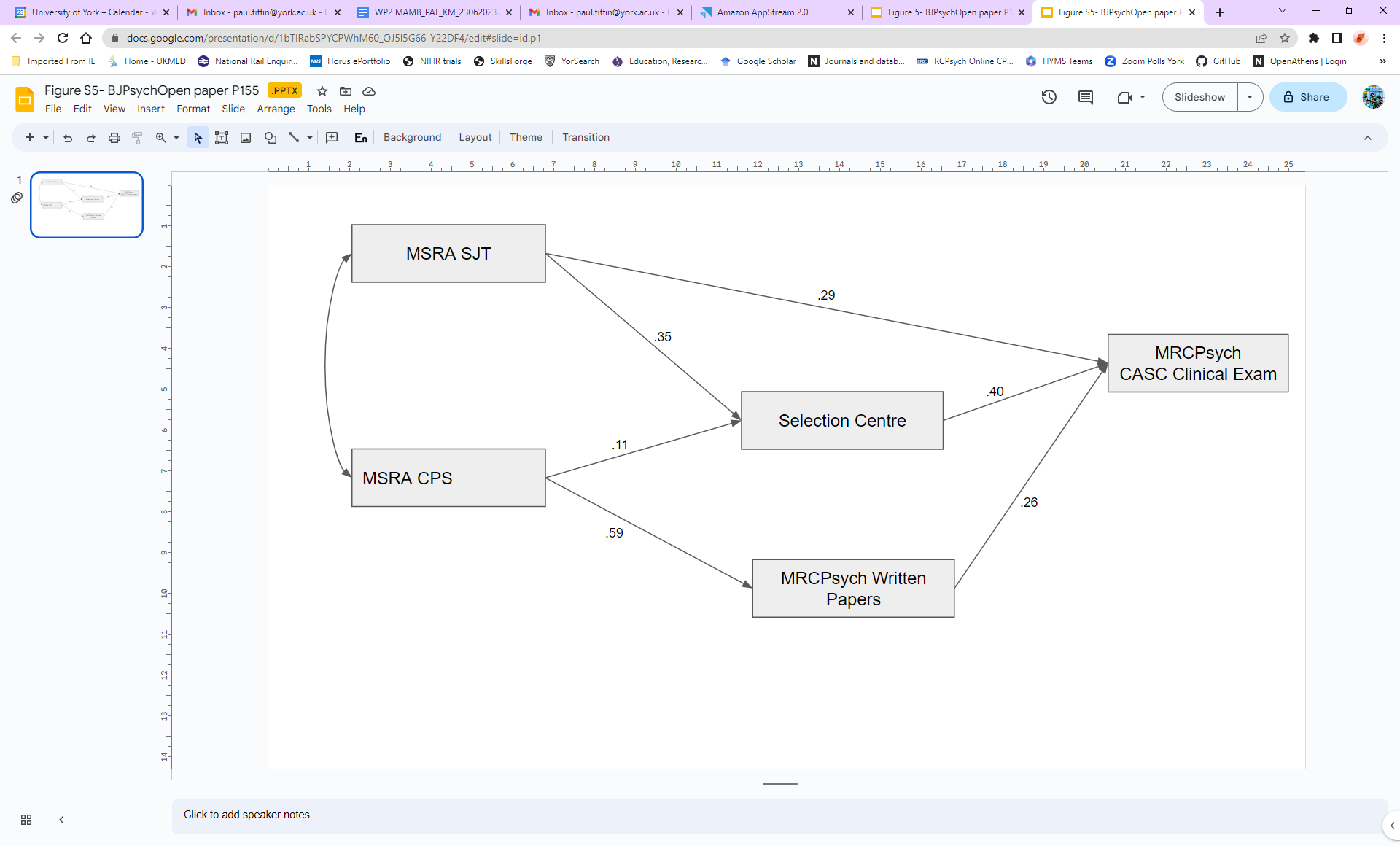


Figure S6. Results from the estimation of a path model testing the relationship between the predictors and outcome (CASC score) in the non-imputed dataset (N=2,015*).

*Note that all observations were included in the estimation using FIML except those with missing values for all the dependent variables (SC, Written and CASC, n=1,495).

It can be seen, that, compared to the results from the path model tested in the non-imputed data, the coefficient of the path from the SC to the CASC score is slightly larger in magnitude (0.40 vs 0.33). In contrast the direct effect of MSRA SJT score is slightly smaller (0.29 vs 0.33). However, there is considerable overlap in the confidence intervals for the non-imputed and imputed results, indicating that the differences could still be due to chance alone (e.g. sampling error). For example, the coefficient and confidence intervals for the pathway between the SC and CASC in the non-imputed data is: 0.397 (95% CIs: 0.323 to 0.471). The equivalent values for the model in the imputed data are: 0.331 (0.278 to 0.384).

Two specific potential mediation paths were also evaluated. Firstly, the path from the SJT score to CASC, via SC performance. For the model with all applicants this showed only a modest, though statistically significant effect with a standardised coefficient of 0.12 and a p value of <0.001. This indicates that only the minority of the predictive ability of the SJT is mediated via SC performance. Similarly, it was shown that a modest proportion of the ability of the CPS scores to predict eventual CASC performance was mediated via the average written MRCPsych examination scores. In this respect the standardised mediational effect estimated in the modified model with the imputed data was 0.15, p<0.001). For the low scorer model these values were 0.07 (p<0.001) and 0.09 (p<0.001) respectively. This indicated very small, albeit statistically significant mediational effects in these regards.

**References**

1. Royal College of Psychiatrists. Preparing for exams London: Royal College of Psychiatrists; 2019 [Available from: https://www.rcpsych.ac.uk/training/exams/preparing-for-exams ].

2. Royal College of Psychiatrists. CASC guide for candidates London: Royal College of Psychiatrists; 2019 [Available from: https://www.rcpsych.ac.uk/training/exams/preparing-for-exams/casc-guide-for-trainees ].

3. Kramer A, Muijtjens A, Jansen K, Dusman H, Tan L, van der Vleuten C. Comparison of a rational and an empirical standard setting procedure for an OSCE. Objective structured clinical examinations. *Medical Education*. 2003;37(2):132-9.4.

4. Botan V, Williams N, Law GR, Siriwardena AN. How is performance at selection to general practice related to performance at the endpoint of GP training?: University of Lincoln; 2022.

5. Taylor C, McManus I, Davison I. Would changing the selection process for GP trainees stem the workforce crisis? A cohort study using multiple-imputation and simulation. *BMC Medical Education*. 2018;18(1):1-7.

6. Lievens F, Sackett PR, De Corte W. Weighting admission scores to balance predictiveness-diversity: The Pareto-optimization approach. *Medical Education*. 2022;56(2):151-8.
